# Supplementary material for: Y RNA fragment in extracellular vesicles confers cardioprotection via modulation of IL‐10 expression and secretion
Source: EMBO Mol Med. 2017 Feb 6;9(3):337–52. doi: 10.15252/emmm.201606924 (PMC5331234; doi:10.15252/emmm.201606924)
Supplement: Supplementary file 1 — Appendix [file EMMM-9-337-s001.pdf]

Table of contents

Appendix Figure S1. CDC-EVs and NHDF-EVs size/concentration.
Appendix Figure S2. CDCs potency from different donors *in vivo.*
Appendix Figure S3. EV-YF1 transfer in control NHDFs compared to CDCs.
Appendix Figure S4. EV-YF1 modulates IL-10 expression.
Appendix Figure S5. EV-YF1 induced direct protection of cardiomyocytes from oxidative stress.
Appendix Figure S6. Cardiac *Il10* and EV-YF1 expression after I/R.
Appendix Figure S7. EV-YF1 reduced the number of inflammatory macrophages CD68+ and apoptotic cardiomyocytes in scar area.
Appendix Figure S8. Exosome isolation protocol.
Appendix Figure Legends.

Appendix Figure S1. CDC-EVs and NHDF-EVs size/concentration.

A

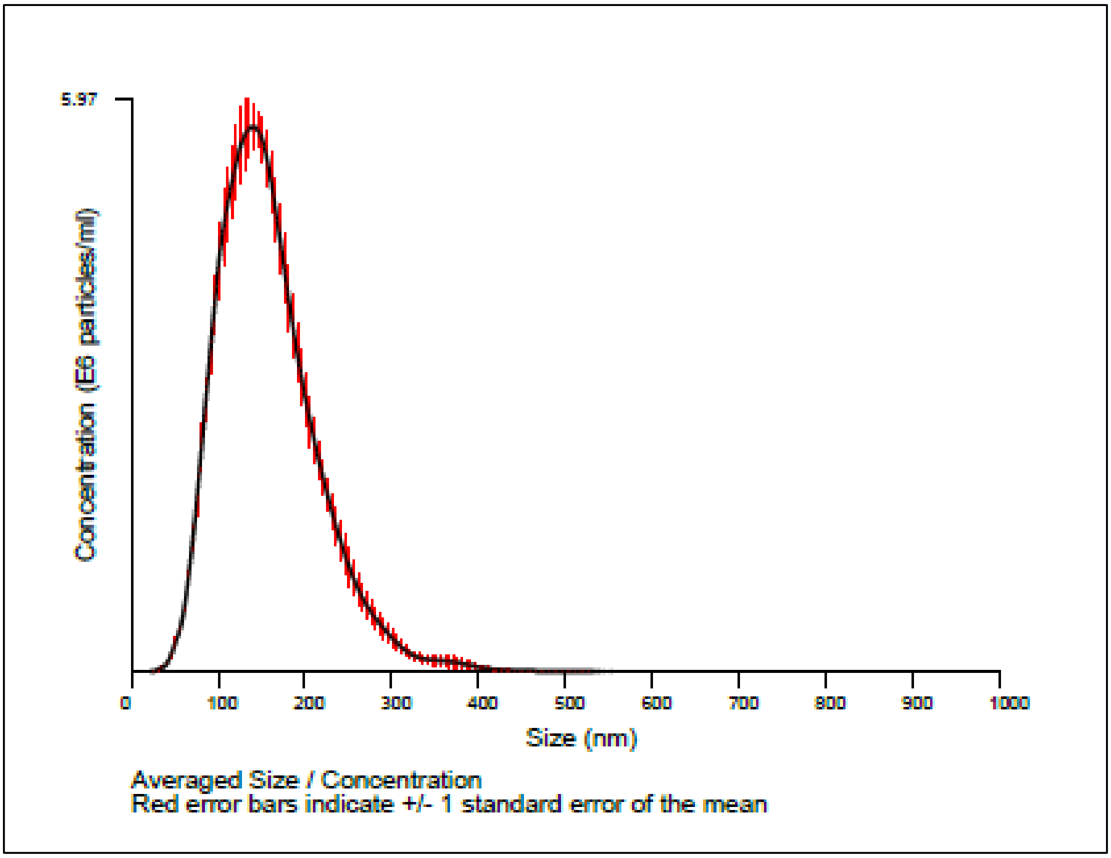

B

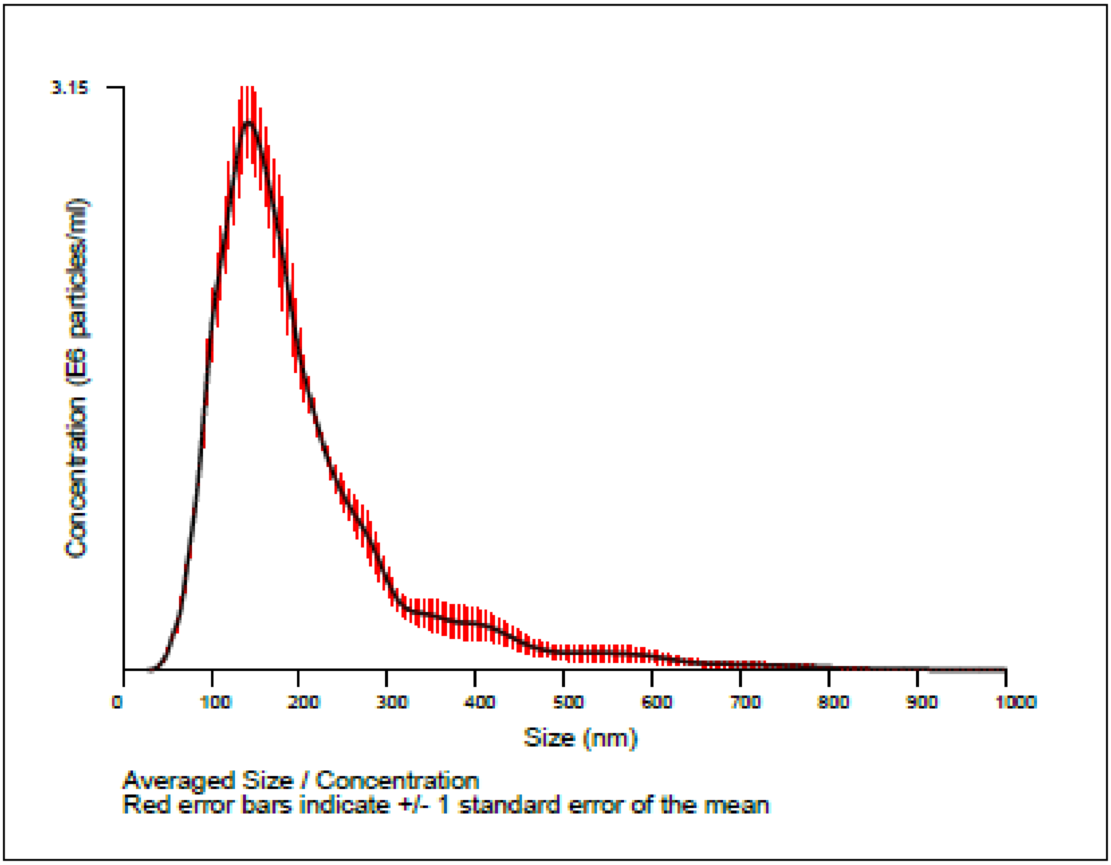

Appendix Figure S2. CDCs potency from different donors *in vivo*.

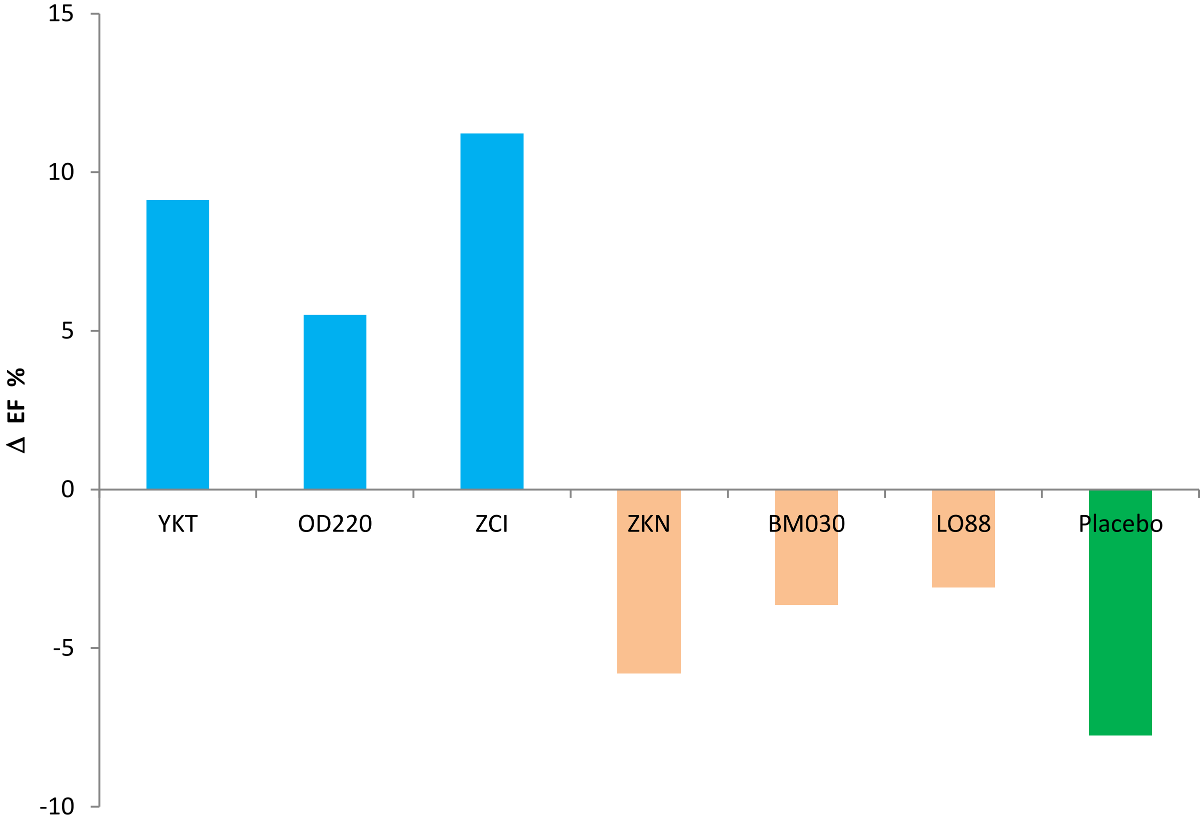

Appendix Figure S3. EV-YF1 transfer in control NHDFs compared to CDCs.

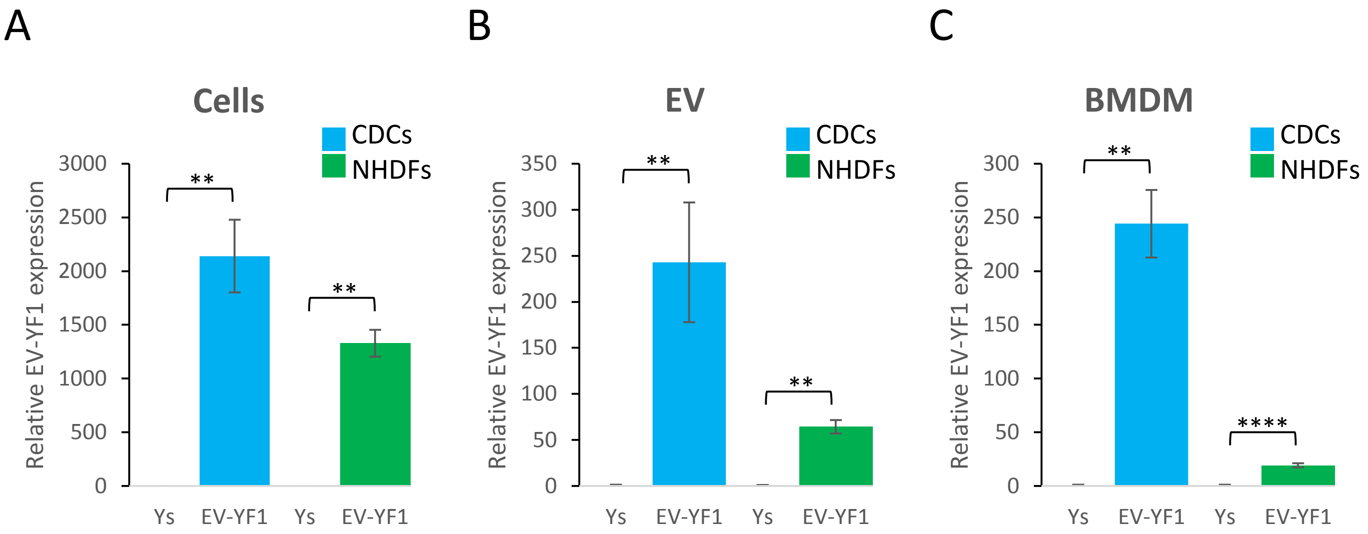

Appendix Figure S4. EV-YF1 modulates IL-10 expression.

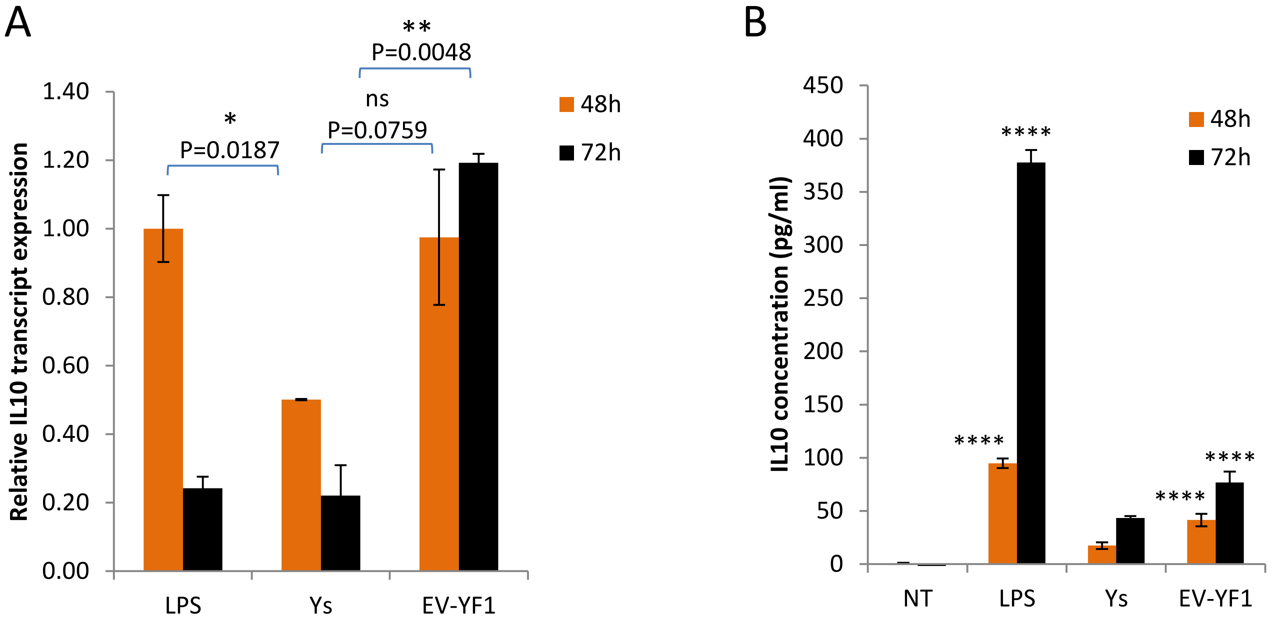

Appendix Figure S5. EV-YF1 induced direct protection of cardiomyocytes from oxidative stress.

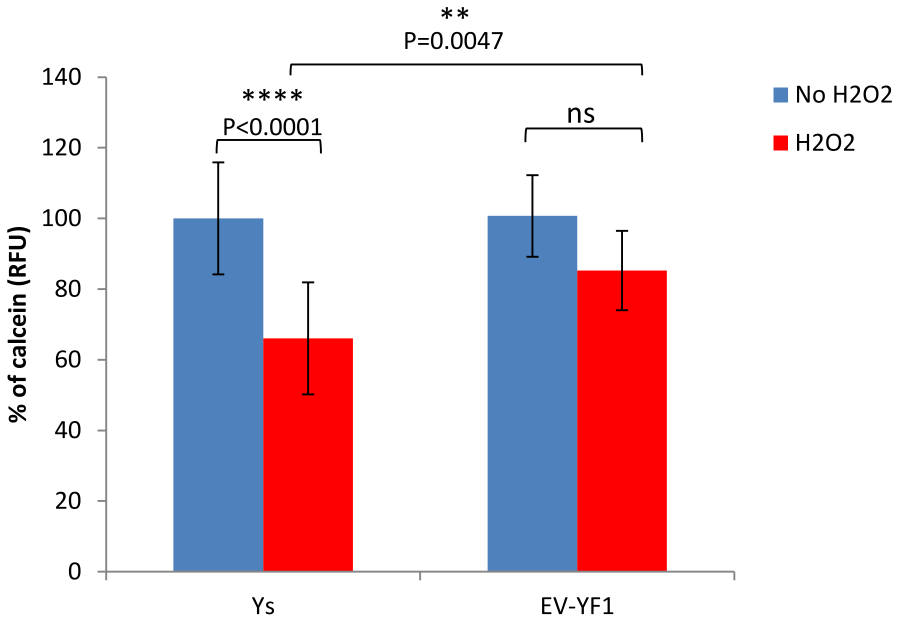

Appendix Figure S6. Cardiac *Il10* and EV-YF1 expression after I/R.

A

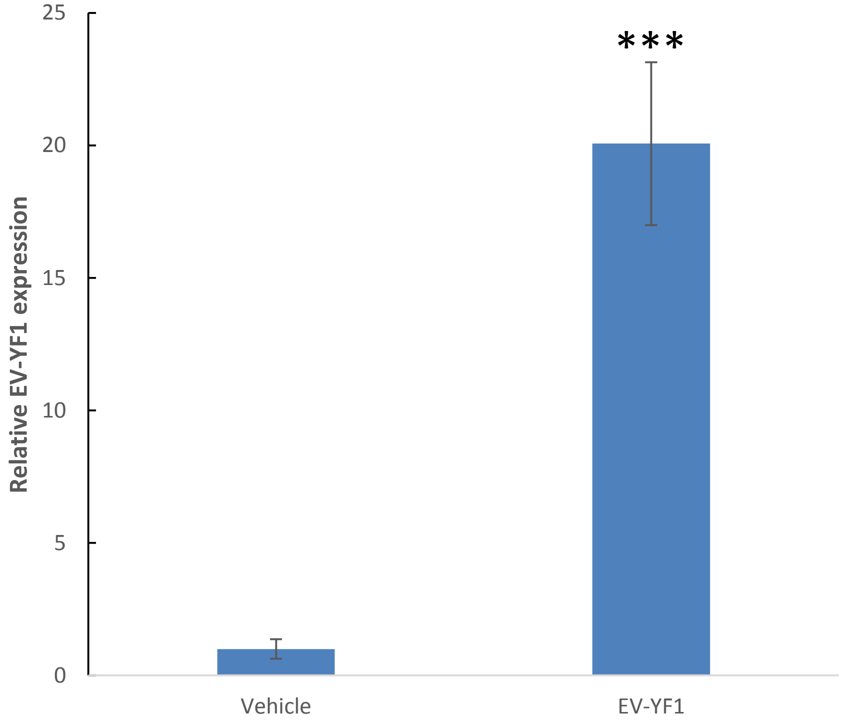

B

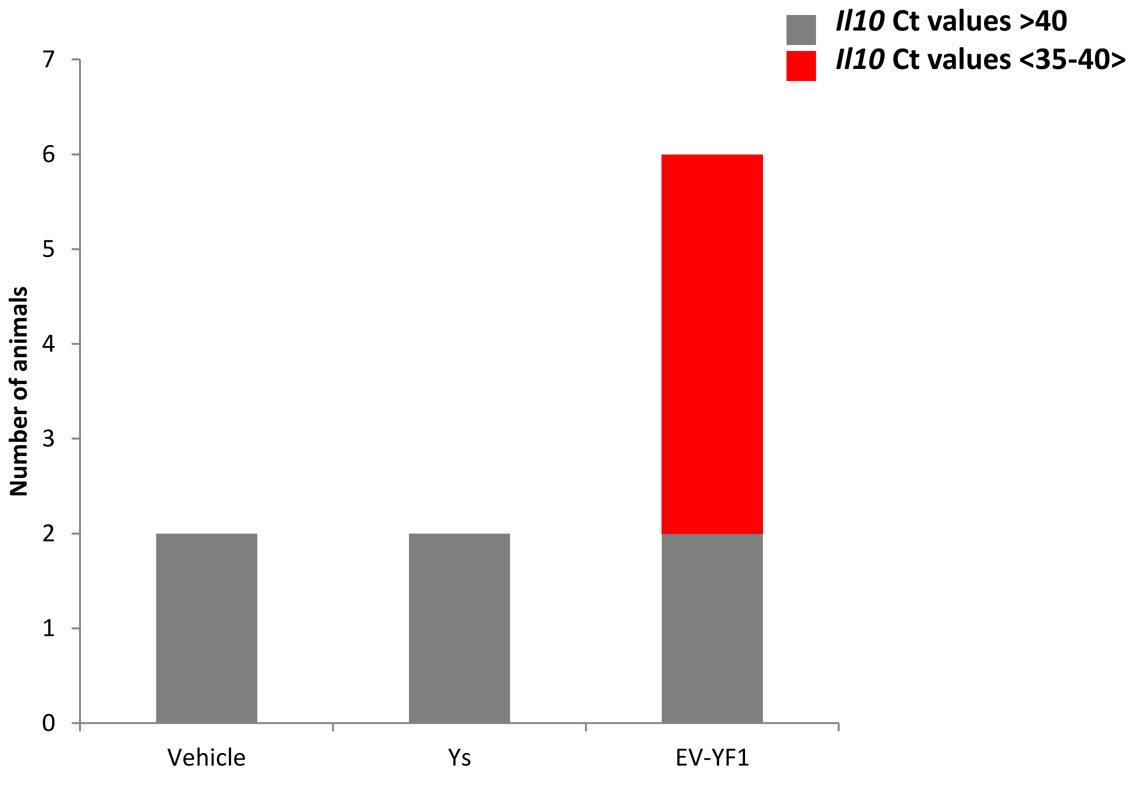

Appendix Figure S7. EV-YF1 reduced the number of inflammatory macrophages CD68+ and apoptotic cardiomyocytes in scar area.

A

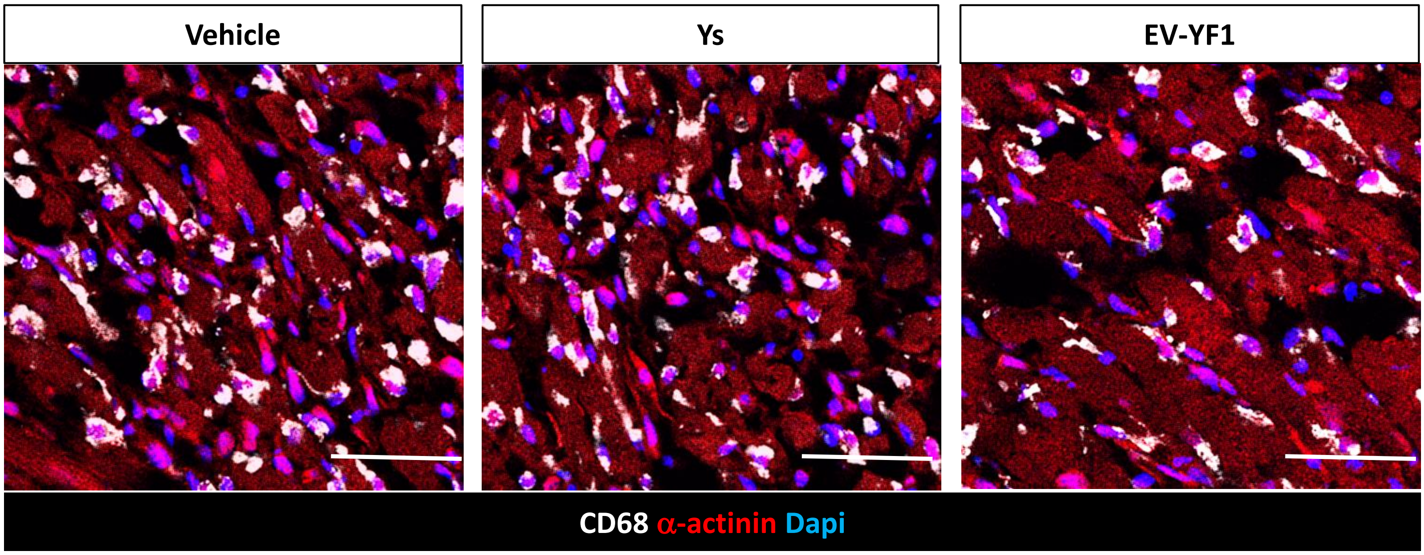

B

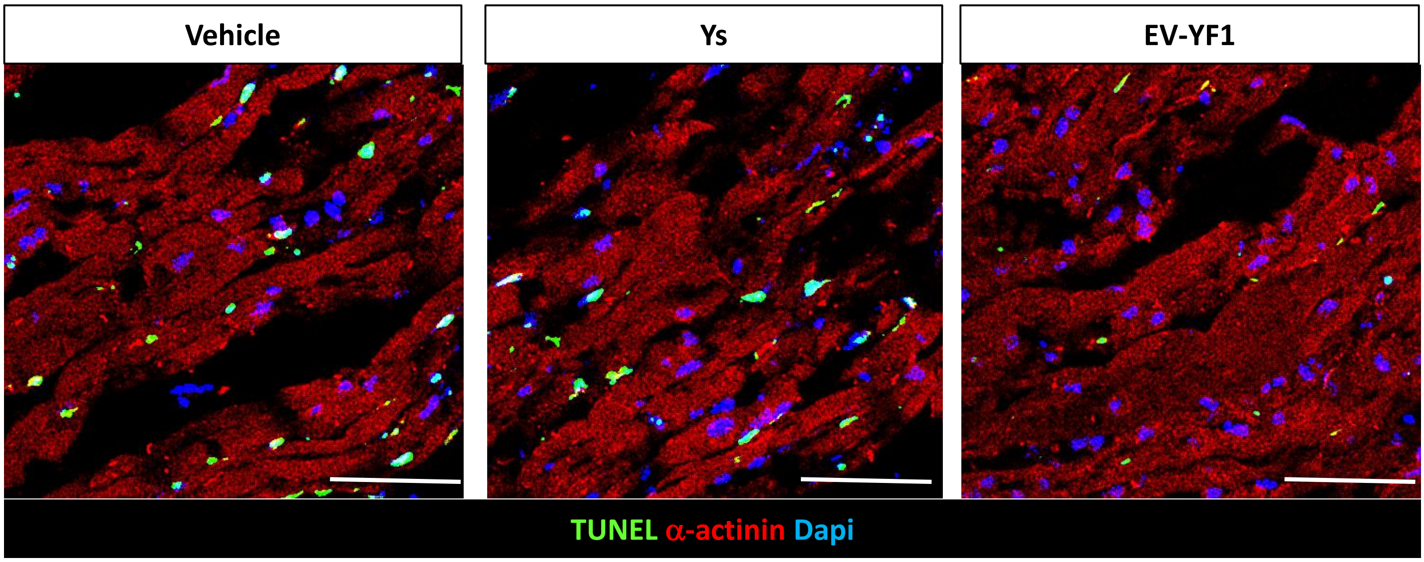

Appendix Figure S8. Exosome isolation protocol.

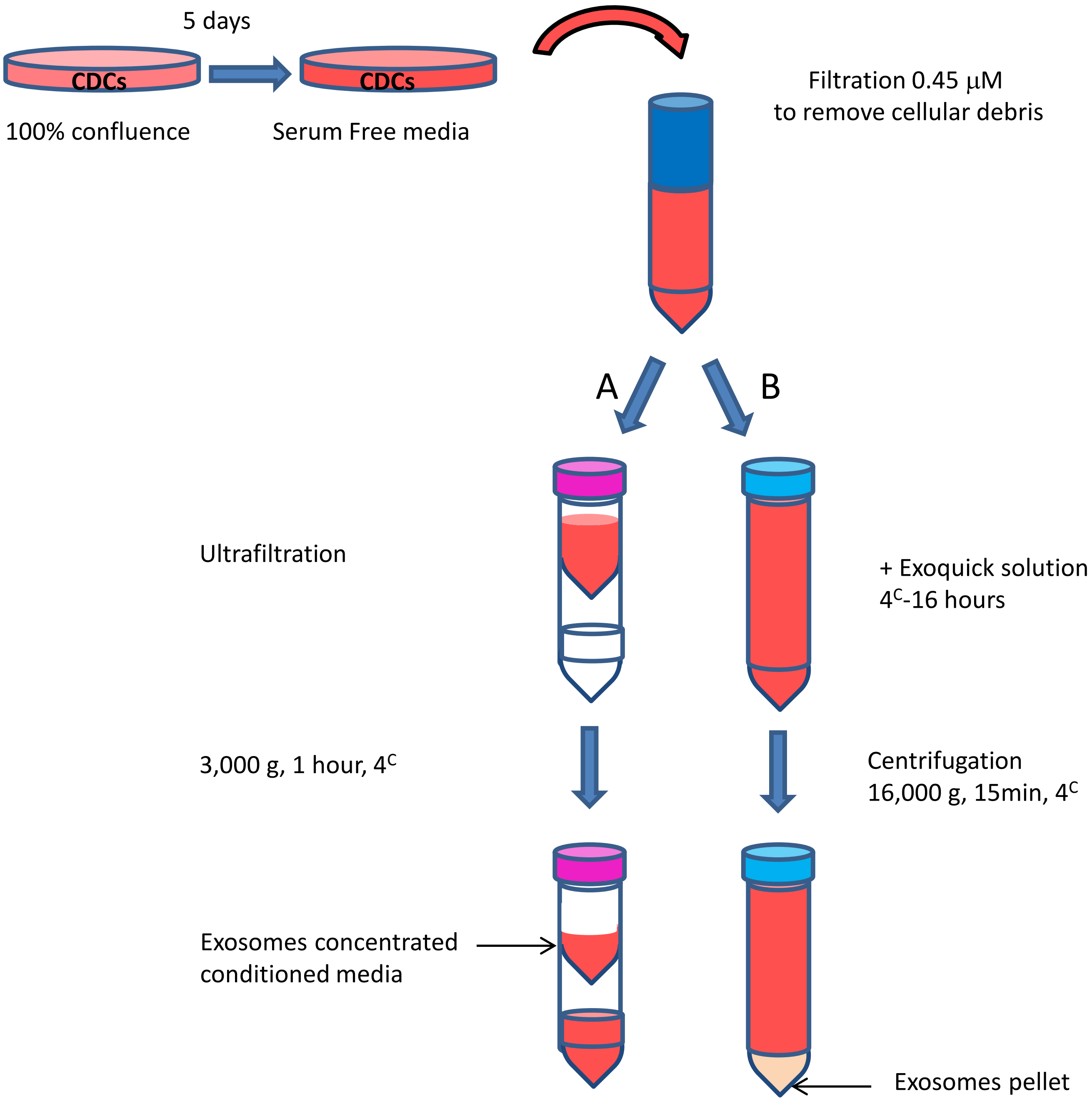

APPENDIX FIGURE LEGEND

Appendix Figure S1 - CDC-EVs and NHDF-EVs size/concentration.

A, B CDC-EVs (A) and NHDF-EVs (B) size distribution and particle number were analyzed by the LM10-HS system (NanoSight). Histogram of CDC-EVs diameter, representative of results from a total of 6 donors.

Appendix Figure S2 - CDCs potency from different donors *in vivo*.

Percent change in ejection fraction (baseline 2 hrs post-MI to 21 days, ΔEF%) post-MI with CDC treatment (6 different donors, n=8 animals/donor) or placebo (saline, n=14 animals). Potent CDCs (ZCI, YKT, OD220) were delineated from non-potent CDCs (LO88, BM030, ZKN) by positive ΔEF%.

Appendix Figure S3. EV-YF1 transfer in control NHDFs compare to CDCs.

A, B, C Expression of EV-YF1 by qPCR in cells (A), EVs (B) and BMDMs (C) according the same protocol described in Figure 4A for CDCs (blue) and NHDFs (green). Results depict the mean ± SEM of n=3. (Groups were compared using 2-tailed, unpaired, Student's t test; (A) (CDC: $p=0.0013$; NHDF: $p=0.0088$), (B) (CDC: $p=0.0059$; NHDF: $p=0.0068$) and (C) (CDC: $p=0.0019$; NHDF: $p<0.0001$).

Appendix Figure S4 – EV-YF1 modulates IL-10 expression.

A *Il10* gene expression in BMDMs at 48 and 72 hrs after treatment with LPS ([1µg/ml]; positive control) or transfection with EV-YF1 or Ys. Results depict the mean ± SEM of two independent experiments, n=6. (Groups were compared using 1-way ANOVA followed by Tukey's multiple comparisons test).

B IL-10 protein secretion from conditioned media in (A), as determined by ELISA. Results depict the mean ± SEM of an experiment representative of two independent experiments, n=6. (Groups were compared using 1-way ANOVA followed by Tukey's multiple comparisons test; ****$p<0.0001$).

Appendix Figure S5 - EV-YF1 induced direct protection of cardiomyocytes from oxidative stress. Graph depicts viability of NRVMs overexpressing EV-YF1 (or Ys) undergoing oxidative stress. NRVMs were transfected with EV-YF1 (or Ys) 48 hrs prior 15 min of H_2O_2 (75uM) treatment, then viability was assessed after 6 hrs by calcein staining. Results depict the mean ± SEM of three independent experiments in triplicate, n=9. (Groups were compared using 1-way ANOVA followed by Tukey's multiple comparisons test).

Appendix Figure S6 - Cardiac *Il10* and EV-YF1 expression after I/R.

A EV-YF1 expression was assessed in ischemic/reperfused heart by qPCR one hour after injection. Results depict the mean ± SEM of two animals per group. (Groups were compared using 2-tailed, unpaired, Student's t test; ***$p=0.0007$).

B *Il10* gene expression in hearts of animals treated with EV-YF1, Ys or vehicle 24h after I/R. Only animals treated with EV-YF1 revealed *Il10* expression (Ct values between 35 and 40); no *Il10* expression was detected in heart treated with Ys or vehicle (Ct values >40).

Appendix Figure S7. EV-YF1 reduced the number of inflammatory macrophages CD68+ in scar area.

Representative immunohistochemical images of the infarct tissue from animals treated with vehicle, Ys, or EV-YF1.

A Macrophage infiltration (Fig 7D). CD68 (white), α-actinin (green), and DAPI (blue). Scale bar: 10μm.

B TUNEL analysis (Fig 7E). TUNEL (green), α-actinin (red), and DAPI (blue). Scale bar: 10μm.

Appendix Figure S8 - Exosome isolation protocol.

A Exosomes-enriched EVs fraction concentrated from conditioned media are used to treat cells directly or after transfection of exosomes.

B Exosomes-enriched EVs fraction pellet submitted to RNA-seq.
